# Psoas muscle attenuation on preoperative CT predicts 30-day complications after lateral lumbar interbody fusion

**DOI:** 10.1016/j.bas.2026.106063

**Published:** 2026-04-24

**Authors:** Julien N. Jost, Kristina Catalano, Pascal Luterbacher, Jaqueline Lattmann, Debora Cipriani, Lukas Andereggen, Gerrit A. Schubert, Markus Bruder

**Affiliations:** a Department of Neurosurgery, Cantonal Hospital Aarau, Aarau, Switzerland; b Faculty of Medicine, University Hospital of Bern, Bern, Switzerland; c Department of Neurosurgery, RWTH Aachen University, Aachen, Germany; d Department of Neurosurgery, Goethe University, Frankfurt am Main, Germany; e Department of Neuroradiology, Cantonal Hospital Aarau, Aarau, Switzerland

**Keywords:** Lateral lumbar interbody fusion, Psoas attenuation, Myosteatosis, Sarcopenia, Postoperative complications, Risk stratification

## Abstract

**Introduction:**

Computed tomography (CT)–derived mean psoas muscle attenuation (P-HU) reflects myosteatosis and has been associated with frailty-related outcomes. Whether it predicts early postoperative complications after lateral lumbar interbody fusion (LLIF) remains unclear.

**Research question:**

Does preoperative P-HU predict 30-day postoperative complications, prolonged hospitalization, and approach-related hip flexion weakness after LLIF?

**Material and methods:**

We retrospectively analyzed 60 adults undergoing LLIF. Mean bilateral P-HU was measured at mid-L3 on routine preoperative CT. The primary endpoint was any 30-day postoperative complication classified according to Clavien–Dindo (CD ≥ I); clinically relevant complications (CD ≥ II) were evaluated in sensitivity analyses. Multivariable Firth logistic regression adjusted for age, ASA classification, and body mass index. Interobserver reliability was assessed in a subset of 36 patients.

**Results:**

Sixteen patients (26.7%) developed 30-day postoperative complications. P-HU was significantly lower in patients with complications than in those without (35.1 ± 6.0 vs 44.4 ± 7.3 HU; p < 0.001). Each 10-HU increase in P-HU was independently associated with lower odds of complications (OR 0.20; p = 0.002). Discrimination was high (AUC 0.87). Higher P-HU was also associated with shorter hospital stay but not with postoperative hip flexion weakness.

**Discussion and conclusion:**

Lower preoperative P-HU was independently associated with postoperative complications and prolonged hospitalization after LLIF, but not with approach-related hip flexion weakness. Opportunistic CT-based muscle assessment may represent a simple imaging biomarker for perioperative risk stratification.

## Highlights

• Psoas muscle attenuation predicts 30-day complications after LLIF.
• CT-derived muscle quality reflects systemic physiological reserve.
• Lower attenuation is associated with longer hospital stay.
• Muscle attenuation does not predict hip flexion weakness.
• Opportunistic CT analysis may improve perioperative risk stratification.

## Abbreviations

ASA –: American Society of Anesthesiologists classification
BMI –: Body mass index
CD –: Clavien–Dindo classification
COPD –: Chronic obstructive pulmonary disease
CRP –: C-reactive protein
CSA –: Cross-sectional area
CT –: Computed tomography
HU –: Hounsfield units
ICC –: Intraclass correlation coefficient
ICU –: Intensive care unit
IQR –: Interquartile range
LLIF –: Lateral lumbar interbody fusion
LOS –: Length of stay
MRI –: Magnetic resonance imaging
mFI-5 –: Modified Frailty Index-5
MRC –: Medical Research Council
OR –: Odds ratio
P-HU –: Mean bilateral psoas muscle attenuation measured in Hounsfield units
ROC –: Receiver operating characteristic
ROI –: region of interest
SD –: Standard deviation

## 1 Introduction

Lateral lumbar interbody fusion (LLIF) has become an established surgical approach for degenerative lumbar spine disease. By using a transpsoas corridor, LLIF enables indirect decompression and restoration of spinal alignment while minimizing posterior muscle disruption. However, the procedure carries a distinct complication profile. In addition to general perioperative complications, approach-related neurological deficits—particularly transient hip flexion weakness—remain a well-recognized concern (Hijji et al., 2017).

Preoperative risk stratification in spine surgery is traditionally based on demographic and comorbidity-based indices such as age, body mass index (BMI), and American Society of Anesthesiologists (ASA) classification. While these measures capture medical comorbidity, they may not adequately reflect biological reserve or physiological resilience to surgical stress. In recent years, imaging-derived markers of muscle quality have gained increasing attention as potential indicators of frailty and surgical vulnerability (Semonche et al., 2025; Hirase et al., 2021).

Computed tomography (CT)–derived muscle attenuation reflects intramuscular fat infiltration (myosteatosis) and has been associated with adverse postoperative outcomes across several surgical specialties. Lower attenuation values indicate poorer muscle quality and may reflect impaired metabolic and inflammatory resilience. In spine surgery, previous studies have primarily focused on muscle cross-sectional area or sarcopenia indices, whereas quantitative muscle attenuation has received comparatively less attention (Hirase et al., 2025; Hou et al., 2024; Uyeda et al., 2023; Aubrey et al., 2014; Goodpaster et al., 2000).

Complications after LLIF can be broadly divided into systemic perioperative complications and approach-related neurological deficits, particularly hip flexor weakness caused by transpsoas retraction. Whether imaging-based markers of muscle quality primarily reflect vulnerability to systemic complications or also influence approach-related neurological deficits remains unclear. Clarifying this distinction may improve risk stratification and clinical interpretation of CT-derived muscle biomarkers in spine surgery (Li et al., 2021).

## 2 Methods

### 2.1 Study design and patient population

This retrospective cohort study included 60 consecutive adult patients who underwent LLIF for degenerative lumbar spine disease at a tertiary neurosurgical center between January 2022 and February 2025. Eligible patients were ≥18 years of age and had a minimum clinical follow-up of 30 days. Both standard LLIF procedures and hybrid approaches—including anterior lumbar interbody fusion performed in the lateral decubitus position (xALIF)—were included. xALIF procedures were included because they were performed in the same lateral positioning and shared the same perioperative workflow. No other fusion techniques were part of this cohort. All patients underwent supplemental posterior instrumentation with pedicle screw fixation, with additional direct decompression performed only when clinically indicated. Surgical indications comprised degenerative disc disease, lumbar spinal stenosis, and degenerative spondylolisthesis. Patients undergoing surgery for trauma, infection, and tumors were excluded.

Ethical approval for the study was obtained from the Ethikkommission Nordwest-und Zentralschweiz (2024-01216). This study utilized a retrospective design and involved the analysis of existing medical data. All patients included in the study had previously provided general consent for the use of their de-identified medical information for research purposes.

### 2.2 Clinical data collection

Demographic and perioperative data were extracted from electronic medical records, including age, sex, BMI, ASA class, operative time, and number of treated levels. Frailty was assessed using the modified Frailty Index-5 (mFI-5), scored from 0 to 5 according to established criteria (Yagi et al., 2019). Preoperative CT imaging was routinely obtained in all patients undergoing LLIF at our institution during the study period. Postoperative complications were recorded during the index hospitalization and within the first 30 postoperative days based on electronic medical records and routine postoperative follow-up documentation. Complications were graded according to the Clavien–Dindo classification (CD) (Clavien et al., 2009). The primary endpoint was any 30-day postoperative complication graded according to CD. Because CD grade I events may represent minor deviations from the expected postoperative course, sensitivity analyses restricted to clinically relevant complications (CD ≥ II) were performed. Major complications (CD ≥ III) were analyzed descriptively because of the limited number of events. As an additional sensitivity analysis accounting for surgical invasiveness and baseline physiological reserve, we extended the primary Firth penalized logistic regression model by including preoperative hemoglobin, albumin, and the number of LLIF levels as a marker of complexity.

Secondary endpoints included: Length of hospital stay (LOS), measured in days, Discharge to inpatient rehabilitation (defined strictly as transfer to a structured inpatient rehabilitation facility; transitional care or nursing facilities were not considered rehabilitation), and new postoperative hip flexion weakness, defined as Medical Research Council (MRC) grade <5. Motor strength was assessed preoperatively, on postoperative day 1, at discharge, at 4–6 weeks follow-up, and at final follow-up. The relationship between CT-derived mean psoas attenuation (P-HU) and frailty (mFI-5) was explored using correlation analysis (Spearman).

### 2.3 Radiological assessment

**Fig. 1:**
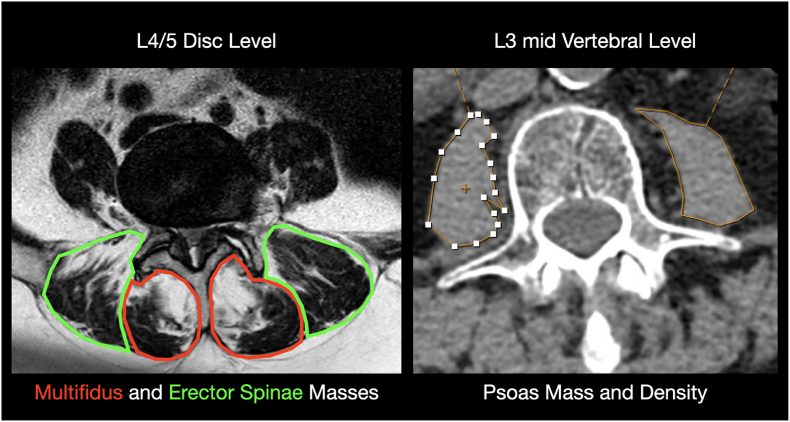
Radiographic Measurement Protocol for Quantitative Muscle Assessment Axial cross-sectional imaging used for quantitative muscle assessment. Left: MRI at the L4–L5 disc level demonstrating manual segmentation of the multifidus (red) and erector spinae (green) muscles for cross-sectional area measurement. Right: CT at the mid-L3 vertebral level illustrating bilateral manual segmentation of the psoas muscles. P-HU was calculated within the outlined region of interest to quantify muscle quality.

Measurements were performed on routine CT scans obtained during standard preoperative imaging (Fig. 1). P-HU and cross-sectional area (CSA, mm^2^) were measured bilaterally at the mid-vertebral level of L3 on axial reconstructions aligned parallel to the endplate using region-of-interest (ROI) techniques. Primary measurements were performed by a board-certified neurosurgeon. For interobserver assessment, measurements were independently repeated by a board-certified neuroradiologist and a neurosurgical resident trained in the standardized ROI protocol in a random subset of 36 patients (60%), both blinded to outcomes and prior measurements. Values from both sides were averaged to obtain a single mean value per patient. Muscle attenuation was used as a surrogate marker of myosteatosis, with lower HU values indicating increased intramuscular fat infiltration and reduced muscle quality. Intramuscular fat was included within the ROI, whereas cortical bone, vascular structures, and adjacent extramuscular fat were excluded. Agreement between the measurements was assessed using the intraclass correlation coefficient (ICC) based on a two-way random-effects model with absolute agreement. In addition, Pearson correlation and Bland–Altman analysis were performed to evaluate systematic measurement differences between observers.

Paraspinal muscle measurements were obtained from preoperative magnetic resonance imaging (MRI). Cross-sectional areas (CSA, mm^2^) of the multifidus and erector spinae muscles were measured bilaterally at the L4–L5 disc level. Multifidus fatty infiltration was graded semi-quantitatively as grade 0 (minimal or no fat), grade 1 (≤25% fat), grade 2 (25–50% fat), and grade 3 (>50% fat infiltration).

### 2.4 Statistical analysis

Continuous variables were assessed for normality using the Shapiro–Wilk test and are presented as mean ± standard deviation or median with interquartile range (IQR), as appropriate. Categorical variables are reported as counts and percentages. Group comparisons were performed using Student's *t*-test or Mann–Whitney *U* test for continuous variables and chi-square or Fisher's exact test for categorical variables.

For the primary endpoint (any 30-day postoperative complication), multivariable Firth penalized logistic regression was performed including P-HU (per 10 HU increase), age, ASA classification (modeled as an ordinal variable), and BMI. Given the limited number of complication events (n = 16), covariates were restricted to a small set of clinically established variables to reduce overfitting. LLIF levels were modeled as a linear count variable (range 1–4). As a sensitivity analysis, ASA was replaced by the modified Frailty Index-5 (mFI-5). Odds ratios (OR) with 95% confidence intervals are reported.

Receiver operating characteristic (ROC) analysis was used to evaluate the discriminative performance of P-HU, with the optimal cutoff determined using the Youden index. LOS showed a right-skewed distribution and was analyzed using log-transformed linear regression; exponentiated coefficients are presented as multiplicative LOS factors. Because all patients with postoperative complications were discharged to inpatient rehabilitation, modeling rehabilitation discharge in the full cohort resulted in complete separation. Therefore, multivariable Firth logistic regression for rehabilitation discharge was restricted to patients without in-hospital complications.

As a sensitivity analysis, the primary regression model was repeated using clinically relevant complications defined as CD ≥ II. This analysis was performed to evaluate whether the association between P-HU and 30-day postoperative complications persisted when minor complications were excluded. Given the limited number of outcome events, the number of covariates in the primary model was intentionally restricted to established clinical risk factors to reduce the risk of model overfitting.

Model performance was additionally assessed using the Brier score and calibration metrics (intercept and slope). A two-sided p-value <0.05 was considered statistically significant. All statistical analyses and graphical visualizations were performed using R (R Foundation for Statistical Computing, Vienna, Austria).

## 3 Results

### 3.1 Patient characteristics

**Fig. 2:**
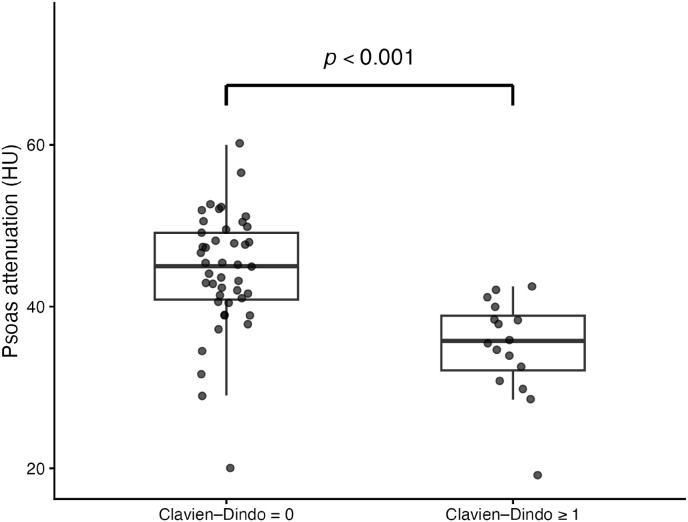
Distribution of Psoas Muscle Attenuation According to 30-day postoperative complication status Distribution of P-HU measured at the L3 level in patients with and without any 30-day postoperative complication (Clavien–Dindo ≥ I). Boxplots represent median and interquartile range; whiskers indicate 1.5 × interquartile range. Individual observations are shown as jittered points. Lower attenuation values were observed in patients who developed postoperative complications.

Sixty consecutive patients undergoing LLIF for degenerative lumbar spine disease were included in the analysis. The median follow-up was 9 months (IQR 3–15). 30-day postoperative complications occurred in 16 patients (26.7%). The mean age of the cohort was 67.5 ± 9.4 years and the mean BMI was 28.9 ± 4.9 kg/m^2^. Thirty patients (50.0%) were classified as ASA ≥ III. Lower preoperative albumin levels were observed in patients who developed postoperative complications (35.7 ± 5.4 g/L vs 39.2 ± 5.0 g/L), although this difference did not reach statistical significance. The mean psoas muscle attenuation (P-HU) was 41.9 ± 8.1 HU. Patients who developed complications (CD ≥ I) had significantly lower preoperative P-HU compared with those without complications (35.1 ± 6.0 HU vs 44.4 ± 7.3 HU, p < 0.001) (Fig. 2). Baseline demographic, clinical, and surgical characteristics stratified by complication status are summarized in Table 1. According to the CD classification, 6 complications were grade I, 7 were grade II, and 3 were grade ≥ III. Grade I complications included wound healing disorders, electrolyte disturbances (hypokalemia or hyperkalemia), postoperative nausea and vomiting, pressure ulcer formation, and increased postoperative pain requiring intensified pain management or physiotherapy. Grade II complications comprised pneumonia requiring antibiotic therapy, delirium requiring pharmacological treatment and intermediate care monitoring, deep vein thrombosis treated with anticoagulation, initiation of antiplatelet therapy following a transient ischemic attack, electrolyte disturbances requiring medical treatment, and cases requiring erythrocyte concentrate transfusion. Major complications (CD ≥ III) occurred in three patients and included percutaneous seroma drainage without general anesthesia (grade IIIa) and surgical wound revision for postoperative infection under general anesthesia (grade IIIb) (Table 2).

**Table 1:** Baseline demographic, clinical, and surgical characteristics stratified by 30-day postoperative complications (Clavien–Dindo ≥ I).

Variable	Total (n = 60)	CD = 0 (n = 44)	CD ≥ I (n = 16)	p-value
Age (years), mean ± SD	67.5 ± 9.4	66.7 ± 9.9	69.9 ± 7.7	0.14
Female sex, n (%)	28 (46.7%)	18 (40.9%)	10 (62.5%)	0.16
BMI (kg/m^2^), mean ± SD	28.9 ± 4.9	28.8 ± 4.6	29.3 ± 5.9	0.73
ASA class, median (IQR)	2 (2–3)	2 (2–3)	3 (2–3)	0.08
mFI-5, mean ± SD	1.0 ± 0.8	1.0 ± 0.8	1.2 ± 0.8	0.31
Diabetes, n (%)	13 (21.7%)	9 (20.5%)	4 (25.0%)	0.73
Hypertension, n (%)	36 (60.0%)	25 (56.8%)	11 (68.8%)	0.55
Chronic heart failure, n (%)	4 (6.7%)	2 (4.5%)	2 (12.5%)	0.29
COPD, n (%)	8 (13.3%)	6 (13.6%)	2 (12.5%)	1.0
Smoking history, n (%)	38 (63.3%)	29 (65.9%)	9 (56.2%)	0.55
Anticoagulation, n (%)	18 (30.0%)	12 (27.3%)	6 (37.5%)	0.53
Preoperative hemoglobin, mean ± SD	137.6 ± 16.8	140.4 ± 13.9	129.9 ± 21.8	**0.02**
Preoperative albumin, mean ± SD	38.1 ± 5.3	39.2 ± 5.0	35.7 ± 5.4	0.08
Preoperative CRP, median (IQR)	2 (1–4)	1 (1–3)	3 (1–4)	0.46
Revision surgery, n (%)	36 (60.0%)	24 (54.5%)	12 (75.0%)	0.23
L4/5 level included, n (%)	21 (35.0%)	14 (31.8%)	7 (43.8%)	0.54
Hybrid procedure (xALIF L5/S1), n (%)	10 (16.7%)	6 (13.6%)	4 (25.0%)	0.43
Operative time (min), mean ± SD	256.9 ± 112.3	244.0 ± 92.9	292.4 ± 151.7	0.58
Estimated blood loss (mL), median (IQR)	200 (150–400)	200 (150–400)	250 (175–550)	0.32
LLIF levels, median (IQR)	1 (1–2)	1 (1–2)	2 (1–2)	**0.01**
Lumbar Cobb angle (degrees), mean ± SD	13.7 ± 8.8	12.8 ± 8.5	16.0 ± 9.5	0.3
Psoas attenuation (HU), mean ± SD	41.9 ± 8.1	44.4 ± 7.3	35.1 ± 6.0	**<0.001**
Psoas CSA total (mm^2^), mean ± SD	1040.7 ± 548.0	1069.8 ± 603.0	960.8 ± 360.1	0.91
Multifidus CSA total (mm^2^), mean ± SD	2275.9 ± 698.0	2368.1 ± 688.9	2022.4 ± 679.9	0.24
Erector spinae CSA total (mm^2^), mean ± SD	2429.4 ± 672.5	2520.1 ± 720.9	2179.9 ± 445.6	0.06
Paraspinal CSA total (mm^2^), mean ± SD	4705.3 ± 1208.9	4888.2 ± 1221.7	4202.3 ± 1049.8	0.08
Multifidus fat infiltration grade, median (IQR)	2 (1–3)	2 (1–3)	2 (2–3)	0.01

**Table 2:** Postoperative outcomes stratified by 30-day postoperative complications (Clavien–Dindo ≥ I).

Variable	Total (n = 60)	CD = 0 (n = 44)	CD ≥ I (n = 16)	p-value
LOS (days), median (IQR)	5 (3–7)	4 (3–5)	8 (6–8)	**<0.001**
Discharge to inpatient rehabilitation, n (%)	22 (36.7%)	7 (15.9%)	15 (93.8%)	**<0.001**
ICU admission, n (%)	2 (3.3%)	0 (0.0%)	2 (12.5%)	0.07
30-day readmission, n (%)	6 (10.0%)	1 (2.3%)	5 (31.2%)	**0.004**
30-day reoperation, n (%)	2 (3.3%)	0 (0.0%)	2 (12.5%)	0.07

### 3.2 Association between P-HU and complications

**Fig. 3:**
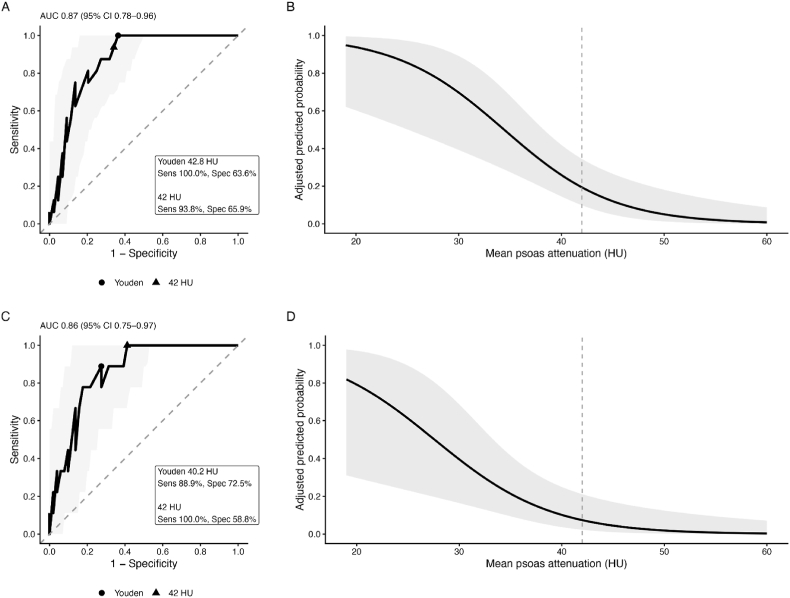
Predictive Performance and Clinical Interpretation of P-HU for Postoperative Complications A: ROC analysis predicting any 30-day postoperative complication (Clavien–Dindo ≥ I). B: Predicted probability of complications across P-HU values adjusted for age, ASA classification, and BMI. C: ROC analysis predicting clinically relevant complications (Clavien–Dindo ≥ II). D: Adjusted predicted probability for clinically relevant complications.

ROC analysis demonstrated strong discriminative performance of P-HU (Fig. 3) for predicting 30-day postoperative complications (AUC 0.87; 95% CI 0.78–0.96). When stratified by the ROC-derived threshold of 42 HU, complication rates differed markedly between groups. Patients with P-HU ≤42 HU had a substantially higher 30-day postoperative complication rate compared with those with attenuation >42 HU (50.0% vs 3.3%, p < 0.001). The cutoff should be interpreted as exploratory because it was derived from the study cohort. P-HU showed only a weak and non-significant correlation with the modified Frailty Index-5 (Spearman ρ = −0.21, p = 0.11). The direction and magnitude of the association remained consistent when measurements were repeated by an independent, blinded neuroradiologist, supporting the robustness of the imaging-based muscle quality assessment.

### 3.3 Multivariable analysis

In the primary multivariable Firth penalized logistic regression model adjusted for age, ASA classification, and BMI (Table 3), higher P-HU was independently associated with reduced odds of 30-day postoperative complications. Each 10-HU increase in P-HU was associated with an approximately 80% reduction in the odds of complications (OR 0.20; 95% CI 0.06–0.49; p = 0.002). Age, ASA classification, and BMI were not independently associated with complications in the adjusted model.

**Table 3:** Multivariable Firth logistic regression predicting 30-day postoperative complications.

Primary analysis (CD ≥ I)			
Variable	OR	95% CI	p-value
P-HU (per 10 HU)	0.20	0.06–0.49	**0.002**
Age (per year)	0.98	0.90–1.06	0.61
ASA (per category increase)	2.78	0.66–13.41	0.17
BMI (per kg/m^2^)	1.00	0.87–1.13	0.93

Sensitivity analysis (CD ≥ II)			
Variable	OR	95% CI	p-value
P-HU (per 10 HU)	0.17	0.05–0.60	**0.005**
Age	0.99	0.90–1.09	0.83
ASA	4.19	0.49–35.71	0.19
BMI	0.92	0.76–1.11	0.38

**Table 4:** Multivariable log-linear regression analysis for length of hospital stay.

Variable	LOS Factor	95% CI	p-value
P-HU (per 10 HU)	0.76	0.65–0.88	**<0.001**
Age	1.01	0.99–1.03	0.28
ASA	1.18	0.93–1.49	0.16
BMI	1.00	0.98–1.03	0.82

When P-HU was dichotomized using the ROC-derived threshold of 42 HU, low attenuation (≤42 HU) remained strongly associated with complications (adjusted OR 21.0; 95% CI 4.1–238.0; p < 0.001).

In a sensitivity analysis including preoperative hemoglobin and the number of LLIF levels, P-HU remained independently associated with lower odds of complications (OR 0.25 per 10 HU; 95% CI 0.09–0.69; p = 0.007) (Table 5). Alternative models incorporating multifidus fatty infiltration yielded similar findings (Table 6).

**Table 5:** Sensitivity analyses of the multivariable logistic regression model.

Extended Analysis including Hemoglobin and LLIF Levels (Case Complexity)			
Variable	OR	95% CI	p
P-HU (per 10 HU)	0.25	0.09–0.69	**0.007**
Age (per year)	1.01	0.91–1.11	0.91
ASA (per category)	2.77	0.51–15.08	0.24
BMI (per kg/m^2^)	1.06	0.91–1.24	0.44
Preoperative hemoglobin (per unit)	0.97	0.92–1.01	0.13
LLIF levels (per +1 level)	3.15	1.15–8.64	**0.03**

Sensitivity analysis including Albumin as a nutritional marker			
Variable	OR	95% CI	p-value
P-HU (per 10 HU)	0.23	0.08 – 0.70	**0.01**
Age (per year)	1.02	0.92 – 1.13	0.67
ASA (per category increase)	2.41	0.46 – 12.72	0.30
BMI (per kg/m^2^)	1.05	0.90 – 1.23	0.53
Preoperative albumin (per 1 g/L)	0.86	0.72 – 1.04	0.11

**Table 6:** Alternative multivariable model using multifidus fat infiltration instead of P-HU.

Variable	OR	95% CI	p
Multifidus fat infiltration	2.77	1.18–6.49	**0.02**
Age	1.00	0.90–1.10	0.88
ASA	2.36	0.55–10.12	0.25
BMI	1.04	0.94–1.15	0.44
Combined model			
P-HU	0.27	0.10–0.73	**0.01**
Multifidus fat infiltration	2.13	0.80–5.66	0.13

In a complete-case sensitivity analysis additionally adjusting for preoperative serum albumin (n = 46), P-HU remained independently associated with 30-day postoperative complications (OR 0.23 per 10 HU; 95% CI 0.075–0.70; p = 0.010). Preoperative albumin showed a non-significant trend toward lower complication risk (OR 0.86 per 1 g/L; 95% CI 0.72–1.04; p = 0.112).

In a sensitivity analysis restricted to clinically relevant complications (CD ≥ II), events occurred exclusively in patients with P-HU ≤42 HU (30.0% vs 0.0%, p = 0.0019). Results remained consistent in sensitivity analyses restricted to clinically relevant complications.

### 3.4 LOS

**Fig. 4:**
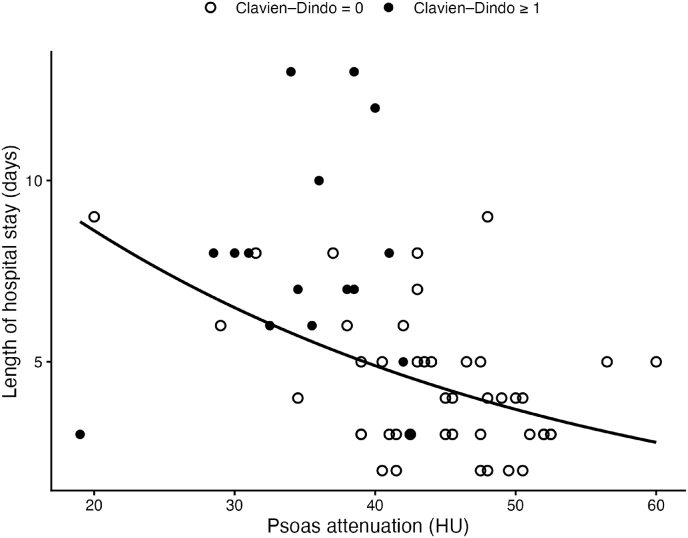
Association Between Psoas Muscle Attenuation and Length of Hospital Stay Scatterplot illustrating the relationship between preoperative mean P-HU and length of hospital stay (LOS). Points are color-coded according to complication status (Clavien–Dindo 0 vs ≥ I). The solid line represents the fitted regression line derived from a log-linear LOS model. Higher P-HU values were associated with shorter hospitalization.

Median LOS was 5 days (IQR 3–7). P-HU correlated inversely with length of stay (Fig. 4) (Spearman ρ = −0.53; p < 0.001). In multivariable log-linear regression analysis adjusted for age, ASA classification, and BMI (Table 4), higher P-HU was independently associated with shorter hospitalization. Each 10-HU increase in attenuation corresponded to a 24% reduction in length of stay (LOS factor 0.76; 95% CI 0.65–0.88; p < 0.001). In a subgroup analysis restricted to patients without 30-day postoperative complications (n = 44), higher muscle attenuation remained independently associated with shorter hospitalization (LOS factor 0.84 per 10 HU; 95% CI 0.73–0.97; p = 0.02).

### 3.5 Discharge to rehabilitation

Twenty-two patients (36.7%) required discharge to inpatient rehabilitation. All patients experiencing 30-day complications during the index hospitalization required postoperative rehabilitation. Among patients without complications, lower P-HU was associated with an increased likelihood of discharge to rehabilitation in univariable analysis, although this association did not remain statistically significant after multivariable adjustment.

### 3.6 Neurological outcomes

**Fig. 5:**
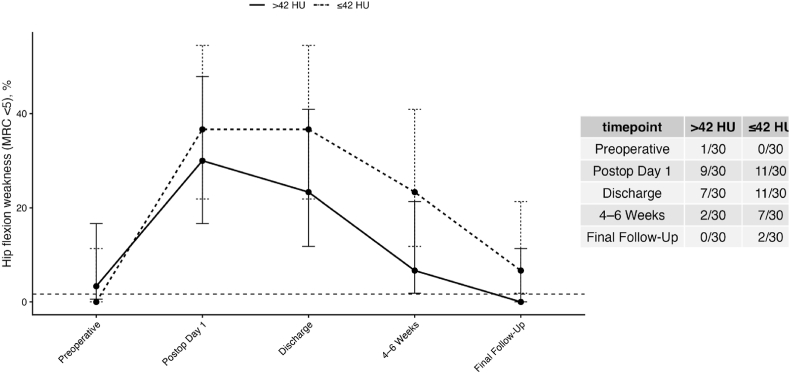
Time Course of Postoperative Hip Flexion Weakness Stratified by Muscle Quality Proportion of patients with hip flexion weakness (Medical Research Council grade <5) over time, stratified by P-HU using a cutoff of 42 HU. Error bars represent 95% Wilson confidence intervals. The dashed horizontal line indicates the baseline preoperative prevalence of weakness. Although descriptive differences in early postoperative weakness were observed, no statistically significant association was identified in adjusted regression analysis.

Postoperative hip flexion weakness occurred in 33% of patients on postoperative day 1 (Fig. 5) and gradually improved during follow-up. Despite the strong association between P-HU and systemic postoperative complications, muscle attenuation showed no association with postoperative hip flexion weakness. In adjusted Firth regression analysis, P-HU was not associated with new postoperative motor weakness (OR per 10 HU 0.93; 95% CI 0.47–1.84; p = 0.99).

### 3.7 Interobserver reliability

Interobserver agreement between neuroradiologist and neurosurgeon measurements demonstrated high reliability (ICC = 0.91) with a Pearson correlation coefficient of r = 0.92. Bland–Altman analysis showed a mean bias of +2.4 HU, with 95% limits of agreement ranging from −3.8 to +8.6 HU, indicating a small systematic tendency toward slightly higher measurements by the surgeon. Exploratory measurements performed by the neurosurgical resident showed comparable attenuation values, supporting the practical reproducibility of the method.

## 4 Discussion

### 4.1 Principal findings

CT-derived psoas muscle attenuation was strongly associated with 30-day postoperative complications following LLIF. Lower attenuation values were associated with substantially higher odds of 30-day postoperative complications and prolonged hospitalization, even after adjustment for established clinical risk factors including age, ASA classification, and BMI. In contrast, muscle attenuation showed no association with postoperative hip flexion weakness. These findings suggest that CT-derived muscle quality likely reflects systemic physiological reserve rather than susceptibility to approach-related neurological injury during transpsoas surgery.

### 4.2 Muscle quality as a marker of physiological reserve

Skeletal muscle attenuation on CT reflects intramuscular fat infiltration (myosteatosis) and has increasingly been recognized as a surrogate marker of frailty and reduced physiological reserve. Lower attenuation values have been associated with impaired immune function, metabolic dysregulation, and diminished capacity to withstand surgical stress in multiple surgical disciplines (Hirase et al., 2021; Hirase et al., 2025; Uyeda et al.; Li et al., 2021; Martin et al., 2013; Camino-Willhuber et al., 2023).

P-HU demonstrated only a weak correlation with the modified Frailty Index-5. This observation supports the interpretation that imaging-derived muscle quality may provide complementary risk information beyond established clinical frailty scores. Opportunistic CT-based assessment may therefore represent a practical adjunct to conventional preoperative risk stratification (Hou et al., 2024; Aubrey et al., 2014).

The association between P-HU and LOS remained significant even with patients without 30-day complications. These findings suggest that muscle quality may influence postoperative recovery trajectories beyond the occurrence of overt complications, potentially reflecting differences in functional reserve and recovery capacity. Hypoalbuminemia is a well-established marker of malnutrition and frailty and has been associated with adverse outcomes after spine surgery. Preoperative albumin levels were lower in patients who developed complications, although the difference did not reach statistical significance. In a sensitivity model additionally adjusting for albumin, P-HU remained independently associated with complications, whereas albumin showed only a non-significant trend. Albumin was evaluated in a secondary exploratory analysis because laboratory data were not available for all patients. This suggests that CT-derived muscle attenuation and serum albumin may reflect related but distinct dimensions of physiological reserve (muscle composition vs nutritional/inflammatory status). (Silva-Fhon et al., 2021).

The present study demonstrated high interobserver reliability between surgeon and blinded neuroradiologist for the measurement of psoas muscle attenuation on routine CT imaging. This finding supports the reproducibility of opportunistic muscle attenuation assessment in clinical practice. The mean measurement difference of 2.4 HU observed in the Bland–Altman analysis suggests minimal systematic bias between observers. Such variability is expected in muscle attenuation measurements and is likely attributable to minor differences in ROI placement, particularly in muscles with varying degrees of fatty infiltration. In addition, exploratory measurements performed by a neurosurgical resident yielded comparable attenuation values, suggesting that opportunistic psoas attenuation assessment may be reproducible even when performed by less experienced observers after brief training.

### 4.3 Distinction between systemic complications and neurological deficits

A notable finding of the present study is the absence of an association between muscle attenuation and postoperative hip flexion weakness. Neurological deficits following LLIF are generally attributed to local mechanical factors inherent to the transpsoas approach, including retraction-related neuropraxia and anatomical variation of the lumbar plexus.

The lack of association between muscle quality and neurological deficit in the present cohort suggests that CT-derived muscle attenuation reflects systemic resilience rather than local susceptibility to surgical corridor injury. This distinction strengthens the biological interpretation of the findings and argues against a nonspecific association between muscle quality and postoperative adverse events (Hijji et al., 2017; Jacob et al., 2022; Pumberger et al., 2012).

### 4.4 Comparison with previous literature

Prior studies evaluating sarcopenia in spine surgery have primarily focused on muscle cross-sectional area or composite sarcopenia indices derived from CT or MRI (Hirase et al., 2021; Charest-Morin et al., 2018).

However, muscle attenuation reflects qualitative changes in muscle composition rather than muscle size alone and may therefore more accurately capture metabolic and physiological vulnerability. Consistent with this concept, previous studies have suggested that CT-derived P-HU may predict postoperative complications after lumbar fusion surgery. Our results support this observation and suggest that muscle quality may represent a more relevant biomarker of systemic surgical risk than simple morphometric muscle measurements.

### 4.5 Clinical implications

These findings have potential implications for perioperative risk stratification in spine surgery. Because CT imaging is frequently obtained during preoperative planning for LLIF, muscle attenuation can be assessed opportunistically without additional cost or radiation exposure.

Identification of patients with low muscle attenuation may help guide perioperative optimization strategies, postoperative monitoring, and discharge planning. Moreover, these findings raise the possibility that targeted prehabilitation or nutritional optimization may improve surgical resilience in vulnerable patients. Prospective studies will be necessary to determine whether modification of muscle quality can translate into improved clinical outcomes (Berardi et al., 2025).

### 4.6 Limitations

Several limitations should be acknowledged. First, the retrospective design introduces the possibility of residual confounding and selection bias. Second, the relatively small sample size and limited number of outcome events represent inherent limitations of the study. To mitigate potential small-sample bias, Firth penalized logistic regression was applied and the number of covariates was intentionally restricted. Third, the attenuation cutoff derived from receiver operating characteristic analysis was generated within the same dataset and should therefore be interpreted as exploratory. External validation in larger cohorts will be required before clinical thresholds can be established. Fourth, inclusion of hybrid lateral-position procedures may have introduced procedural heterogeneity, although all cases shared the same lateral positioning, perioperative workflow, and supplemental posterior instrumentation. Finally, although neurological outcomes were evaluated, the study may have been underpowered to detect subtle associations between muscle quality and approach-related neurological deficits. Because the study was conducted at a single tertiary referral center, external validation in larger multicenter cohorts will be necessary before clinical thresholds can be generalized.

## 5 Conclusions

Preoperative CT-derived P-HU is independently associated with early postoperative complications and prolonged hospitalization following LLIF. This relationship persists after adjustment for established clinical risk factors and likely reflects systemic physiological reserve rather than susceptibility to approach-related neurological injury. Opportunistic assessment of muscle quality on routine preoperative CT imaging may therefore provide a practical tool for perioperative risk stratification in spine surgery.

## Declaration of generative AI and AI-assisted technologies in the manuscript preparation process

During the preparation of this manuscript, the authors used ChatGPT (OpenAI) to assist with language editing and improvement of readability. The authors reviewed and edited all content generated with the assistance of this tool and take full responsibility for the content of the published article.

## Declaration of competing interest

The authors declare that they have no known competing financial interests or personal relationships that could have appeared to influence the work reported in this paper.
